# Effect of different thresholds on the accuracy of linear and volumetric analysis of native- and grafted-bone

**DOI:** 10.1590/0103-6440202204823

**Published:** 2022-08-26

**Authors:** Julia Raulino Lima, Sttephany Silva Bernardino, Lucas De Sousa Goulart Pereira, Túlio Bonna Pignaton, Rubens Spin-Neto, Elcio Marcantonio-Junior, Guilherme José Pimentel Lopes De Oliveira

**Affiliations:** 1 Department of Periodontology, School of Dentistry, Universidade Federal de Uberlândia - UFU, Uberlândia, MG, Brazil; 2 Department of Diagnosis and Surgery, School of Dentistry, Universidade Estadual Paulista - UNESP, Araraquara, SP, Brazil; 3 Department of Dentistry - Oral Radiology Health, Aarhus University, Aarhus, Denmark

**Keywords:** bone graft, histology, tomography, maxillary sinus floor augmentation

## Abstract

The study aimed to evaluate the accuracy of Micro-CT in linear and volumetric measurements in native (NB) and grafted bone (GB) areas. A total of 111 biopsies of maxillary sinuses grafted with deproteinized bovine bone (DBB) in humans were evaluated. The linear measurements were performed to measure the length of the NB and GB. Furthermore, the amount of mineralized tissues at the NB and GB was performed. In the histomorphometry analysis the percentage of mineralized tissues at the NB and GB was obtained in two histological sections while the mineralized tissues were measure in the micro-CT varying the thresholds of the grayscale varying from 90-250 to 90-150 with 10 levels of variation between each one was applied. Then these data were correlated in order to check the higher r level between the histomorphometry and micro-CT thresholds intervals. The linear length of the NB was 2.44±0.91mm and 2.48±1.50mm, respectively, for micro-CT and histomorphometry (r =0.57), while the linear length of the GB was 3.63±1.66mm and 3.13±1.45mm, respectively, for micro-CT and histomorphometry (r =0.74) Histomorphometry showed 45.91±11.69% of bone in NB, and 49.57±5.59% of bone and biomaterial in the GB. The total volume of mineralized tissues that were closest to the histometric analysis were 43.75±15.39% in the NB (Threshold:90-240; r = 0.50) and 51.68±8.42% in the GB (Threshold:90-180; r =-0.028). The micro-CT analysis showed good accuracy in the linear analysis in both portions of the biopsies but for volumetric analysis just in NB.

## Resumo

Esse estudo tem como objetivo avaliar a acurácia da análise microtomográfica em mensurações lineares e volumétricas em osso nativo (ON) e enxertado (OE). Para isso, 111 biópsias removidas de seios maxilares de pacientes enxertados com osso bovino desproteinizado foram coletadas e avaliadas. As medidas lineares foram realizadas para medir o comprimento do ON e do OE. Além disso, foi realizada a mensuração da quantidade de tecidos mineralizados em ON e OE. Na análise histomorfométrica a porcentagem de tecidos mineralizados do ON e OE foi obtida em dois cortes histológicos enquanto os tecidos mineralizados foram medidos em microtomografias variando os thresholds da escala de cinza variando de 90-250 a 90-150 com 10 níveis de variação entre cada. Em seguida, esses dados foram correlacionados para verificar o maior nível de R entre os intervalos dos thresholds testados na análise microtomográfica em relação aos dados obtidos na histomorfometria. O comprimento linear do ON foi de 2,44±0,91mm e 2,48±1,50mm, respectivamente, para análises microtomográfica e histomorfométrica (r=0,57), enquanto o comprimento linear do OE foi de 3,63±1,66mm e 3,13±1,45mm, respectivamente, para para análises microtomográfica e histomorfométrica (r =0,74) A histomorfometria detectou 45,91±11,69% de osso na porção de ON e 49,57±5,59% de osso e biomaterial na porção de OE. O volume total de tecidos mineralizados detectados pela análise microtomográfica que apresentou valores mais próximos da análise histomorfométrica foi de 43,75±15,39% no ON (Thresholds:90-240; r = 0,50) e 51,68±8,42% no OE (Thresholds:90-180; r =- 0,028). A análise microtomográfica apresentou boa acurácia na análise linear em ambas as porções das biópsias, porém a mesma apresentou boa acurácia para análise volumétrica apenas em áreas de ON.

## Introduction

The amount of bone available is an important aspect to be considered during the treatment of edentulism with dental implants ^1^. Because of this, the application of the guided bone regeneration techniques associated with the use of bone grafts in patients with inadequate bone volume for the installation of implants has been widely encouraged ^2^. Among the types of bone substitutes, the autografts have been considered the standard due to the biological superiority of this type of bone graft ^2^ ^,^ ^3^. However, the risk of morbidity at the donor site and the limitation of quantity disponible presented in some cases led to the use of alternative bone substitutes for this purpose ^4^ ^,^ ^5^.

A myriad of alternative osteoconductive bone substitutes have been indicated in order to reduce the necessity of autografts ^6^ ^,^ ^7^. So, the evaluation of the grated areas is essential for the determination of the biological potential of bone formation associated with different osteoconductive bone substitutes ^8^. Among the methods of analysis available for the assessment of the grafted areas, histomorphometry has been considered the gold standard for allowing evaluation of new bone, biomaterial reabsorption, and observation of the interface between graft particles and neoformed bone indicating the osteoconductive capacity of biomaterial predicting the success or failure of the graft for subsequent rehabilitation with dental implants ^6^ ^,^ ^9^ ^,^ ^10^. However, some limitations, such as two-dimensional analysis and the necessity for long periods of descaling to produce histological sections highlight the importance of developing alternative evaluation techniques ^11^.

The tomographic techniques are non-destructive methods that have been used for the evaluation of the grafted areas in clinical studies, spatially for the evaluation of the augmentation of the bone disponible and the stability of bone volume, however, this technique have not enough resolution to distinguish bone and the bone substitutes remnants ^12^. For the study of small samples, such as biopsies obtained in preclinical and clinical studies, microcomputed tomography (micro-CT) has been used as a method of evaluating the structure of grafted areas tridimensional with higher resolution and which theoretically means an occurrence of the lower amount of artifacts in relation to conventional tomography ^11^ ^,^ ^12^. 

Indeed, micro-CT may be a good option to reduce the time and the complexity for the analysis of the grafted areas composition compared with the histomorphometric analysis. However, some parameters may influence the accuracy of the micro-CT analysis in order to detect the mineralized tissues. The analysis of the impact of these parameters is essential in order to check if the micro-CT analysis can be a predictable method to substitute the histomorphometric analysis of the grafted bone tissue. The aim of this study was to investigate the accuracy of the micro-CT in the evaluation of the linear and volumetric areas of native and grafted bone in biopsies of maxillary sinuses grafted with deproteinized bovine bone (DBB) in humans and its correlation with histomorphometric analysis.

## Methodology

### Ethical considerations

This cross-sectional study was carried out with biopsies harvested from the maxillary sinuses of 19 patients that were grafted with deproteinized bovine bone. This study was previously approved by the Ethics Committee of the Faculty of Dentistry of Araraquara (CEP-FOAr, CAAE: 37753514.6.0000.5416) and registered at REBEC (UTN: U1111-1173-9435). The patients were treated between January 2015 to December 2016.

### Biopsies harvest

A total of 111 biopsies of the previously grafted maxillary sinuses were collected from 19 patients, and the patients were considered as a sample unit. These biopsies were collected during the implant placement procedure in the second surgical step, where a 3 mm external diameter trephine replaced the guide drill in order to harvest histological samples from the grafted areas. The use of the trephine drill did not provide any type of additional risk or harm to the treatment and/or the patient's health. All biopsies had a component of native (NB) and grafted bone (GB). The biopsies were fixed in buffered paraformaldehyde 4% for 48 hours and were subsequently kept in 70º alcohol until the time of scanning on the micro-CT scanner

### Micro-CT analysis

**Figure 1:**
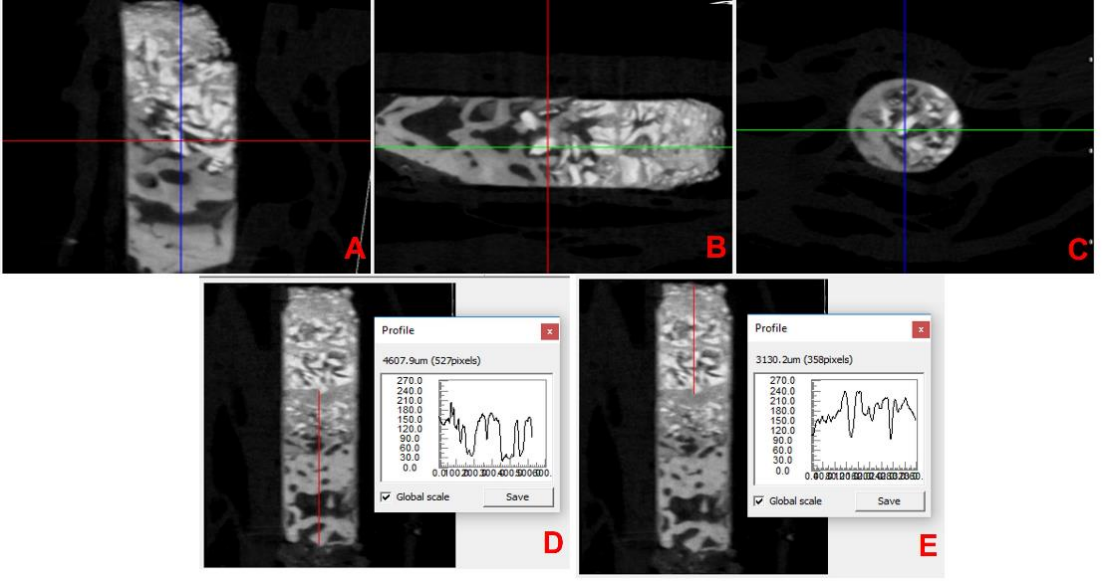
(A-C) Spatially reoriented of the biopsy. The section selected for the analysis was considered to be inside the middle portion of the biopsy in order to be more similar to the region where the histological section was collected. (D-E) The linear length was measured taking into consideration the transition between the NB and GB. After the delimitation of this line, the linear length of the were obtained by the use of the data viewer.

**Figure 2:**
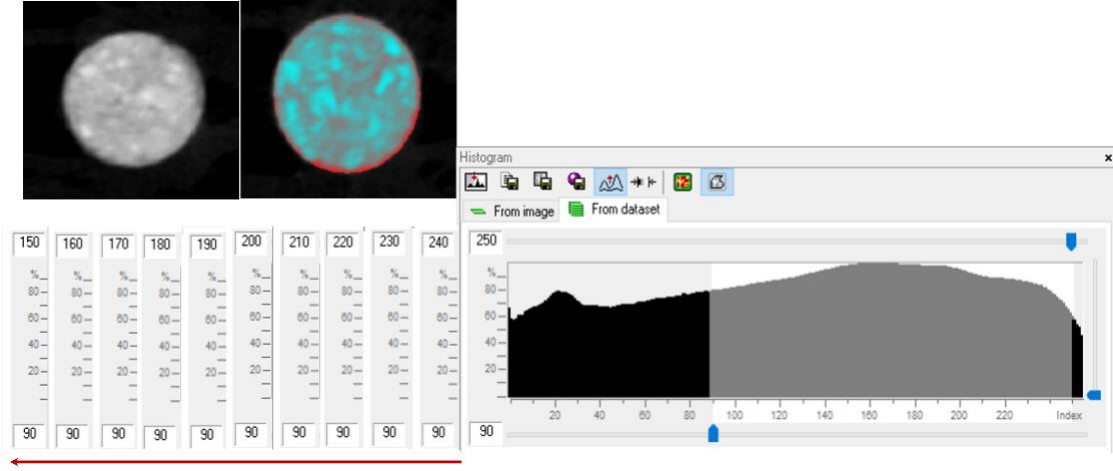
The trans axial images of the samples were used for the analysis of the volume of the mineralized tissues. The entire biopsies were involved in the region of interest. the NB and the GB were evaluated separately, varying the thresholds of the grayscale, in order to distinguish the mineralized tissue from the non-mineralized tissues (90-250; 90-240; 90-230; 90-220; 90-210; 90-200; 90-190; 90-180; 90-170; 90-160, and 90-150).

The biopsies were scanned using the Skyscan device (SkyScan, Kontich, Belgium) with the following parameters: Camera Pixel: 12.45; x-ray tube potential: 65 kVP, x-ray intensity: 385 µA, integration time: 300 ms, filter: Al-1 mm, and voxel size: 18 µm^3^. The generated images were subsequently reconstructed (NRecon, Skyscan, Aartselaar, Belgium), spatially reoriented (DataViewer, Skyscan, Aartselaar, Belgium) and analyzed (CTan, Skyscan, Aartselaar, Belgium) by specific software’s. Linear lengths and the volume of mineralized tissues from NB and GB were measured (Figure 1). The linear length was measured taking into consideration the transition between the NB and GB. After the delimitation of this line, the linear length were obtained by the use of the data viewer. The section selected for the analysis was considered to be inside the middle portion of the biopsy in order to be more similar to the region where the histological section was collected. Specifically in the volumetric evaluation of mineralized tissues, the NB and the GB were evaluated separately, varying the thresholds of the grayscale, in order to distinguish the mineralized tissue from the non-mineralized tissues (90-250; 90-240; 90-230; 90-220; 90-210; 90-200; 90-190; 90-180; 90-170; 90-160, and 90-150) (Figure 2). Then, the volume of the NB and GB was calculated as the BV/TV (%) within these different ranges of the threshold greyscale.

### Sample processing and histologic evaluation

After micro-CT image acquisition, the biopsies were decalcified in buffered ethylenediaminetetraacetic acid (EDTA) 7% for 90 days. The samples were later embedded in paraffin and cut in 5 mm thick cuts that were stained using the Hematoxylin-Eosin technique. Two sections were analyzed per sample with a distance of 40 µm between them, obtained at the center of the biopsy. Images of the histological sections were obtained using an optical microscope (Diastar - Leica eichert & Jung products, Germany), associated with a digital photo camera (DFC-300-FX, Leica Microsystems, Germany) with 1.3-megapixel resolution with an increase of 25X (Figure 1C).

**Figure 3:**
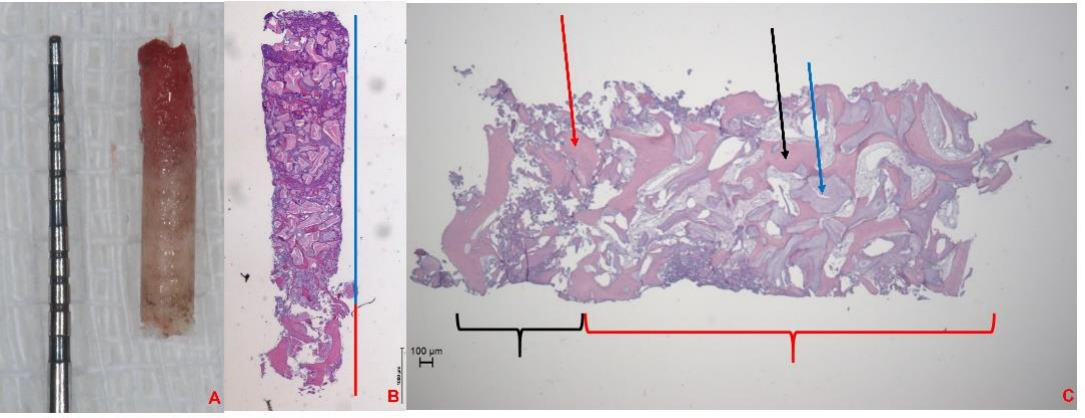
(A) Biopsy sample collected from grafted maxillary sinus. (B) Hematoxylin and Eosin (H&E) stained sections of the biopsy used for histomorphometry analysis. The red line represents the analysis of the linear length of the NB and the blue line represents the measure of the linear length of the GB. (C) The evaluation of the composition of the biopsies at the NB indicated by the black bracket is possible to check only the presence of the bone indicated by the red arrow that was considered the mineralized tissues of these regions. Furthermore, the GB indicated by the red bracket presents the mineralized tissue composed by bone (black arrow) and the DBB remnants (blue arrow).

The histomorphometry was performed with an image analyzer software (Image J, San Rafael, CA, EUA). The linear length of the NB was evaluated from the top of the biopsies to the transition of the NB to the GB, and then the GB was measured from this region to the bottom of the biopsy. The evaluation of the composition of the biopsies were also measured separated between the different compartments. In the part of the biopsy corresponding to the NB, the percentage of mineralized tissues was evaluated in relation to the total area of this portion of the biopsy. In the part corresponding to the GB, the percentage of residual graft material and new bone was evaluated in relation to the total area of the GB (Figure 3). These measurements were correlated with the measurements of the micro-CT analysis.

### Statistical analysis

The data were subjected to the normality test and after normal distribution confirmation, the Pearson's test was performed to assess the correlation between histometric and micro-CT analysis in relation to linear and volumetric analysis (mineralized tissues) in native and grafted areas. The GraphPad Prism 6 software (San Diego, CA, USA) was used for inferential data analysis. All tests were applied at a significance level of 5%.

## Results

### Linear analysis

In the micro-CT analysis, the linear length of the native and grafted bone was 2.59 ± 1.61 mm and 3.63 ± 1.66 mm, respectively. In the histometric analysis, the length of native bone was 2.48 ± 1.50 mm while the length of the grafted bone was 3.13 ± 1.45 mm. The level of correlation between these analyzes was positive and significant (r = 0.578 in native bone and r = 0.743 in grafted area).

### Volumetric analysis

Histometric analysis showed a percentage of bone of 45.91 ± 11.69% in the native bone area, and 49.57 ± 5.59% of bone and biomaterial in the grafted area. The total volume of mineralized tissues that were closest to the values of the histometric analysis were 43.75 ± 15.39% in the native bone area (threshold: 90-240) (Table 1) and 51.68 ± 8.42% in the grafted area (threshold: 90-180) (Table 2). The level of correlation between these analyzes was positive and significant (r = 0.50) in native bone and non-significant (r = -0.028) in the grafted area.

**Table 1:** Mean and standard deviation of the amount of bone and mineralized tissues assessed by histomorphometry and micro-CT in areas of native bone.

Threshold / % Mineralize tissues	BV/TV	%bone	r
90-250	43.67 ± 15.74	45.91 ± 11.69	0.505
90-240	43.75 ± 15.39	45.91 ± 11.69	0.500
90-230	43.38 ± 15.19	45.91 ± 11.69	0.490
90-220	42.80 ± 14.94	45.91 ± 11.69	0.486
90-210	42.03 ± 14.69	45.91 ± 11.69	0.481
90-200	41.25 ± 14.43	45.91 ± 11.69	0.484
90-190	40.20 ± 14.16	45.91 ± 11.69	0.482
90-180	38.60 ± 13.77	45.91 ± 11.69	0.480
90-170	36.49 ± 12.85	45.91 ± 11.69	0.462
90-160	33.42 ± 11.29	45.91 ± 11.69	0.416
90-150	29.33 ± 9.41	45.91 ± 11.69	0.409

**Table 2:** Correlations of BV / TV assessed by the micro-CT analysis and % Bone +% DBB assessed by histomorphometric analysis.

Threshold	BV/TV	%Bone + % DBB	r
90-250	76.31 ± 5.33	50.23 ± 5.13	0.233
90-240	74.64 ± 5.93	50.23 ± 5.13	0.187
90-230	72.19 ± 6.47	50.23 ± 5.13	0.140
90-220	68.96 ± 6.81	50.23 ± 5.13	0.093
90-210	65.24 ± 7.18	50.23 ± 5.13	0.024
90-200	61.28 ± 7.57	50.23 ± 5.13	-0.010
90-190	56.62 ± 8.02	50.23 ± 5.13	-0.017
90-180	51.68 ± 8.42	50.23 ± 5.13	-0.028
90-170	46.24 ± 8.07	50.23 ± 5.13	0.013
90-160	40.61 ± 6.92	50.23 ± 5.13	0.120
90-150	34.91 ± 6.89	50.23 ± 5.13	0.144

## Discussion

Analysis of bone tissue usually requires a longer period for its execution due to the need for histological sections in paraffin, which require a period between 15-90 days for the descaling of the samples depending on the agent used. Image analysis provides the advantage of obtaining results almost immediately. However, the conventional imaging methods used in dentistry do not show to be accurate enough to perform volumetric and segmented analyzes, especially in grafted areas. The purpose of this study to use micro-CT for analysis of bone tissue samples has as a theoretical basis the possible reduction of artifacts due to the higher resolution of the images offered by this equipment. In fact, our study demonstrated that the micro-CT analysis of biopsies removed from maxillary sinuses grafted with Deproteinized Bovine Bone (DBB) was sufficiently accurate to perform linear analyzes in areas of NB and GB and volumetric analysis in NB. However, the separation between the newly formed bone and the remaining bone substitute, as well as the volumetric analysis in grafted areas, was not accurate enough to obtain reliable results.

This study demonstrated that micro-CT allowed the execution of linear analyzes with a high degree of precision, even in areas grafted with DBB that have a radiopaque structure. The artifacts produced by radiopaque structures have already been shown to impair linear analysis of images generated with Cone Beam tomography (CB-CTAN) ^13^ ^,^ ^14^. In fact, this difference between the accuracy of these two instruments in linear analysis in radiopaque structures can be explained by the different ways of obtaining the images as well the differences in the resolution. Images generated by micro-CT (~ 0.18µm) have a higher resolution than images generated by tomographs of the CB-CTAN (~ 1mm). In addition, the generation of CB-CTAN images has been shown to impair the formation of images of structures that are further away from the beam emitted by X-rays ^15^. Therefore, it is suggested that micro-CT is a valuable methodological tool for the analysis of different experimental models, in which linear assessments in bone tissue would be necessary, such as, for example, critical defects in calvaria, periodontal defects, and peri-implant bone level ^16^ ^,^ ^17^.

Another important finding in this study was the ability of micro-CT analysis to predict the volume of bone tissue with a high correlation with the amount of bone tissue observed in the histometric analysis in areas of NB. In fact, these results are encouraging, since the proximity to the grafted area did not seem to have an impact on the quality of bone volume analyzes. In addition, greater threshold intervals used demonstrated a greater correlation with histomorphometric analyzes and the reduction of this interval reduced the relative amount of bone volume in relation to the area of this tissue identified in histomorphometry, possibly by eliminating the bone with lower degrees of mineralization from the count ^18^. In this study, NB was not surgically addressed, unlike what occurred in the grafted area, however, the actual impact of threshold bands on bone tissues with different degrees of maturation remains uncertain. However, micro-CT is in fact an excellent tool for analyzing the quantity of bone in volume as well as for assessing its structure, as has been mentioned in previously published studies ^19^.

In areas grafted with DBB, the micro-CT analysis did not obtain the same level of correlation observed in regions of NB in the volumetric analysis. Previous studies have reported the occurrence of artifacts around radiopaque bodies that interfere with interface analyzes ^20^, such as between bone and dental implant or between bone tissue and different types of biomaterials ^21^ ^,^ ^22^. Although other studies demonstrate results in which there is a separation between radiopaque bone substitutes and host bone tissue, it is likely that the differences between biomaterials, experimental models and evaluated periods justify the differences with the findings of this study ^20^. It is likely that this difficulty in assessing areas grafted with DBB may occur due to the effect of electron beam irradiation of bone substitutes, inducing the calcium cross-linking effect that can impair the analysis at the interfaces of these materials such as with metallic devices ^14^.

This study has some drawbacks that must be taken into account when analyzing our findings. It is worth noting that other variables during scanning can also interfere in the resolution of the images ^23^ and, consequently, in the accuracy of the volumetric analysis in GB and not only the changes in the threshold range. Other factors of interference such as the voxel size, the application of the filters, and Gaussian filtration can alter the resolution, and the presence of artifacts as the ring artifacts and beam hardening effects ^23^. In addition, the findings of this study apply only to biopsies of maxillary sinuses grafted with DBB, as factors such as healing time, structure and composition of the bone substitute, the type of the micro-CT scanner and the experimental model can alter the optimal parameters for biopsy analysis of grafted or native bone tissue.

Finally, the micro-CT analysis showed sufficient accuracy to perform linear analysis on NB and grafted with DBB and volumetric analysis on NB. However, this method of analysis has limitations in the volumetric evaluation in areas grafted with DBB.
